# Genetic relatedness, virulence factors and antibiotics susceptibility pattern of *Vibrio cholerae* isolates from various regions during cholera outbreak in Tanzania

**DOI:** 10.1371/journal.pone.0265868

**Published:** 2022-03-25

**Authors:** Hamza Hamad Matimba, Agricola Joachim, Mucho Michael Mizinduko, Irene Anthony Maseke, Salum Kassim Nyanga, Maria Ezekiely Kelly, Ali Said Nyanga, Janneth Maridadi Mghamba, Mtebe Venance Majigo, Ahmed Abade Mohamed

**Affiliations:** 1 Department of Epidemiology and Biostatistics, Muhimbili University of Health and Allied Sciences, Dar es Salaam, Tanzania; 2 Tanzania Field Epidemiology and Laboratory Training Program, Muhimbili University of Health and Allied Sciences, Dar es Salaam, Tanzania; 3 Department Microbiology and Immunology, Muhimbili University of Health and Allied Sciences, Dar es Salaam, Tanzania; 4 National Health Laboratory, Quality Assurance, and Training Centre, Dar es Salaam, Tanzania; 5 Ministry of Health, Community Development, Gender, Elderly and Children, Dar es Salaam, Tanzania; Nitte University, INDIA

## Abstract

**Background:**

Cholera continues to cause morbidity and mortality in developing countries, including Tanzania. Since August 2015, Tanzania Mainland has experienced cholera outbreaks affecting 26 regions and a 1.6% case fatality rate. The current study determined the virulence factors, genetic relatedness and antimicrobial susceptibility patterns of the *Vibrio cholerae* isolated from different regions in Tanzania.

**Methods:**

A cross-sectional study that involved the genetic characterization of *V*. *cholerae* isolates from eleven regions in Tanzania was carried out. There were 99 *V*. *cholerae* isolates collected between January 2016 and December 2017. The study perfomed a Multi-locus Variable-number tandem-repeat analysis for genetic relatedness and Mismatch Amplification Mutation Analysis polymerase chain reaction for analyzing toxin genes. All the isolates were tested for antimicrobial susceptibility using the Kirby Bauer disk diffusion method. Data were generally analyzed using Microsoft excel, where genetic relatedness was analyzed using eBurst software v3.

**Results:**

All isolates were *V*. *cholerae* O1. Ogawa was the most predominant 97(98%) serotype. Isolates were genetically related with a small genetic diversity and were positive for *ctxA*, *tcpA* El Tor virulence genes. All isolates (100%) were sensitive to doxycycline, trimethoprim-sulphamethoxazole, tetracycline, ceftriaxone, and chloramphenicol, while 87.8% were sensitive to ciprofloxacin. A high resistance rate (100%) was detected towards erythromycin, nalidixic acid, amoxicillin, and ampicillin.

**Conclusion:**

The *V*.*cholerae* O1 serotypes Ogawa, El Tor variant predominantly caused cholera outbreaks in Tanzania with strains clonally related regardless of the place and time of the outbreak. Most of the isolates were susceptible to the antibiotic regimen currently used in Tanzania. The high resistance rate detected for the other common antibiotics calls for continuous antimicrobial susceptibility testing during outbreaks.

## Introduction

Cholera, a disease caused by *V*. *cholerae*, continues to cause morbidity and mortality in low-income countries. Out of 200 serogroups of *V*. *cholerae* reported, only *V*.*cholerae* O1 and O139 are associated with the epidemic [1]. About 1.3–4.0 million cholera cases and 21,000–143,000 deaths occur every year worldwide [2]. Pathogenic *V*. *cholerae* strains contain a cholera toxin prophage that carries the genes encoding for cholera toxin, a vital virulence factor responsible for the typical clinical symptoms of the disease [3].

*V*. *cholerae* O1 serotype commonly exists as Classical and El Tor biotypes [3]. Classical biotype was responsible for most of the epidemic diseases in the past century and was replaced by El Tor biotype, responsible for cholera epidemic in endemic areas. The two biotypes of *V*. *cholerae* O1 are closely related in their O-antigen biosynthetic genes suggesting the genetic hybrids [4]. Variants of *V*. *cholerae* O1 strain that appear to have classical and El Tor traits, generically termed as atypical El Tor biotypes, have been reported elsewhere [5–8].

Since 1974 when the first case of cholera was reported in Tanzania [9], the country has experienced numerous cholera outbreaks resulting in many cases and deaths. Subsequently, from August 2015, cholera cases occured in different regions, including Dar-es-Salaam, Mwanza, Kigoma, Arusha, Pwani, Mara, Dodoma, Katavi, Mbeya, Songwe, and Ruvuma. Throughout this time, the country continued reporting cholera cases. In 2017 approximately 28,089 cholera cases and 452 deaths with a case fatality rate of 1.6% were reported by the Ministry of Health, Community Development, Gender, Elderly and Children (unpublished situational analysis by MoHCDGEC). Hence, this study aimed to determine the genetic relatedness, virulence factors and antimicrobial susceptibility patterns of *V*. *cholerae* strains that caused the outbreak that started from August 2015 onwards.

## Methods

### Study design and setting

A cross-sectional study involving eleven regions that experienced cholera outbreaks from January 2016 to December 2017 was carried out. The regions included were Dar-es-Salaam, Mwanza, Kigoma, Arusha, Pwani, Mara, Dodoma, Katavi, Mbeya, Songwe, and Ruvuma. The study included 99 *V*. *cholerae* isolates collected from 11 out of 26 regions of Tanzania Mainland. Ethical clearance to conduct this study was obtained from the Senate Research and Publications Committee of Muhimbili University of Health and Allied Sciences (MUHAS) in Dar es Salaam, Tanzania. Specimens were collected with permission from the National Health Laboratory Quality Assurance Training Center. The patient’s consent was obtained as part of routine surveillance by MoHCDGEC, in Tanzania. A laboratory identification number was assigned to each specimen to maintain patient confidentiality.

### Isolation and identification of *V*. *cholerae*

Preserved *V*.*cholerae* isolates and fresh stool samples were used in this study. Forty-nine preserved *V*.*cholerae* isolates were revived by inoculating into alkaline peptone water at 35.5°C for 6 hours and then sub-cultured on Thiosulfate-Citrate-Bile salt-Sucrose (TCBS) Agar (Deben Diagnostics Ltd, UK), and incubated aerobically at 35.5°C overnight. Stool samples collected from 50 patients with cholera during the outbreak were also cultured similarly. Isolates were identified by biochemical tests (oxidase and string tests) using pure culture from Tryptone Soya Agar (Deben Diagnostics Ltd, UK). Isolates were serogrouped using *V*. *cholerae* polyvalent O1 and O139 sera (Denka Seiken Ltd-Japan) and serotyped by monovalent antisera Ogawa and Inaba (Denka Seiken Ltd-Japan). The procedure included saline controls to detect auto agglutination [10].

### Deoxyribonucleic acid (DNA) extraction

DNA extraction was performed using Qiagen-QIAamp DNA Mini-Kit (Qiagen, Germantown, MD). Briefly, 5μl of overnight *V*.*cholerae* growth on Luria Bertani (LB) broth culture medium was used as per the Qiagen-DNeasy DNA extraction kit protocol [11]. Extracted *V*. *cholerae* DNA was subjected to Multi-locus variable-number tandem-repeat Analysis (MLVA) for genetic relatedness and Mismatch Amplification Mutation Analysis (MAMA) PCR for toxin genes.

### Multiple-locus variable-number tandem repeat analysis

MLVA analysis was performed for five loci (VC0147, VC0437, VC1650, VCA0171, and VCA0283) of *V*. *cholerae* isolates based on the Variable Numbers of Tandem Repeats (VNTR). Each VNTR locus was amplified using specific primers, as described previously [12, 13]. PCR products were separated, detected, and sized using a 3730xl automatic sequencer using internal lane standards (Liz 600), and the Gene Mapper Software V4 (Applied Biosystems, Foster City, CA, USA) to obtain the allelic size. The number of repeats was calculated at each locus using the Microsoft excel program according to the published formula for a specific locus (e.g., VC0171 formula (size– 265/6) and listed sequentially for the five VNTR loci. Genetic relatedness was considered when genotypes possess identical alleles at four of the five allele in the MLVA genotype for each isolate. Data were analyzed using eBurst software version 3 (http://eburst.mlst.net) [12, 13].

### Mismatch amplification mutation assay-polymerase chain reaction

MAMA-PCR was perfomed to distinguish the cholera toxin B subunit of El Tor and Classical biotypes of *V*. *cholerae* O1. MAMA-PCR designed to detect the nucleotide sequence difference at position 203 of the *ctxB* gene to identify cholera toxin of classical and El Tor biotype was used. A conserved forward primer (Fw-con, 5′-ACTATCTTCAGCATATGCACATGG-3′) and two allele-specific primers, Rv-elt 5′-CCTGGTACTTCTACTTGAAACA-3′) and Rv-cla (5′-CCTGGTACTTCTACTTGAAACG-3′) were used (14). Seventy-nine *V*. *cholerae* strains were subjected to MAMA-PCR to detect the *V*. *cholerae* O1 that harbors classical *ctxB* or El Tor *ctxB* separately. N16961 El Tor biotype and 569B classical biotype were used as reference strains. DNA template for PCR, specific primers, Taq polymerase, and nitrogenous bases were prepared. The automated thermal cycler was programmed as follows: Initial denaturation at 94°C for 2 minutes, followed by 30 cycles of denaturation at 94°C for 30 seconds, annealing at 60°C for 20 seconds, and externsion at 72°C for 25 seconds and a final extension at 72°C for 4 minutes [14, 15].

### Detection of *V*. *cholerae* virulence genes

The isolates were examined for the presence of genes encoding virulence using multiplex PCR. The virulence included *ctxA* responsible for cholera toxin and *tcpA* responsible for toxin-co-regulated pilus specific for El Tor and classical strains [15]. Briefly, initial denaturation was done at 94°C for 5 minutes, followed by 30 cycles consisting of 94°C for 1 minute 30 seconds, 60°C for 1 minute 30 seconds, and 72°C for 1 minute 30 seconds and a final extension step of 72°C for 7 minutes. PCR products were analyzed by electrophoresis in 1% agarose gels, stained with ethidium bromide, visualized under UV light, and recorded with the aid of a gel documentation system (Bio-Rad Laboratories, Hercules, and Ca, USA).

### Antimicrobial susceptibility testing

Antimicrobial susceptibility testing (AST) was performed using Kirby Bauer disk diffusion method according to the Clinical and Laboratory Standards Institute (CLSI) guidelines [16]. The antibiotics tested included; ampicillin (10 μg), amoxicillin (30 μg), ceftriaxone (30 μg), Nalidixic acid (30 μg), ciprofloxacin (5 μg), doxycycline (30 μg), and tetracycline (30 μg). Other antibiotics included were chloramphenicol (30 μg), trimethoprim (1.25 μg)-sulphamethoxazole (23.75 μg), and erythromycin (10 μg). The AST was interpreted according to the CLSI guidelines [16]. *S*. *aureus* (ATCC 25923) for erythromycin and *E*.*coli* (ATCC 25922) for the rest of the antibiotics were used as reference strains for quality control.

### Data analysis

Data was generally analyzed using the Microsoft Excel program, where eBurst software was used to analyze genetic relatedness.

## Results

### Distribution and characterization of *V*.*cholerae* isolates

**Fig 1:**
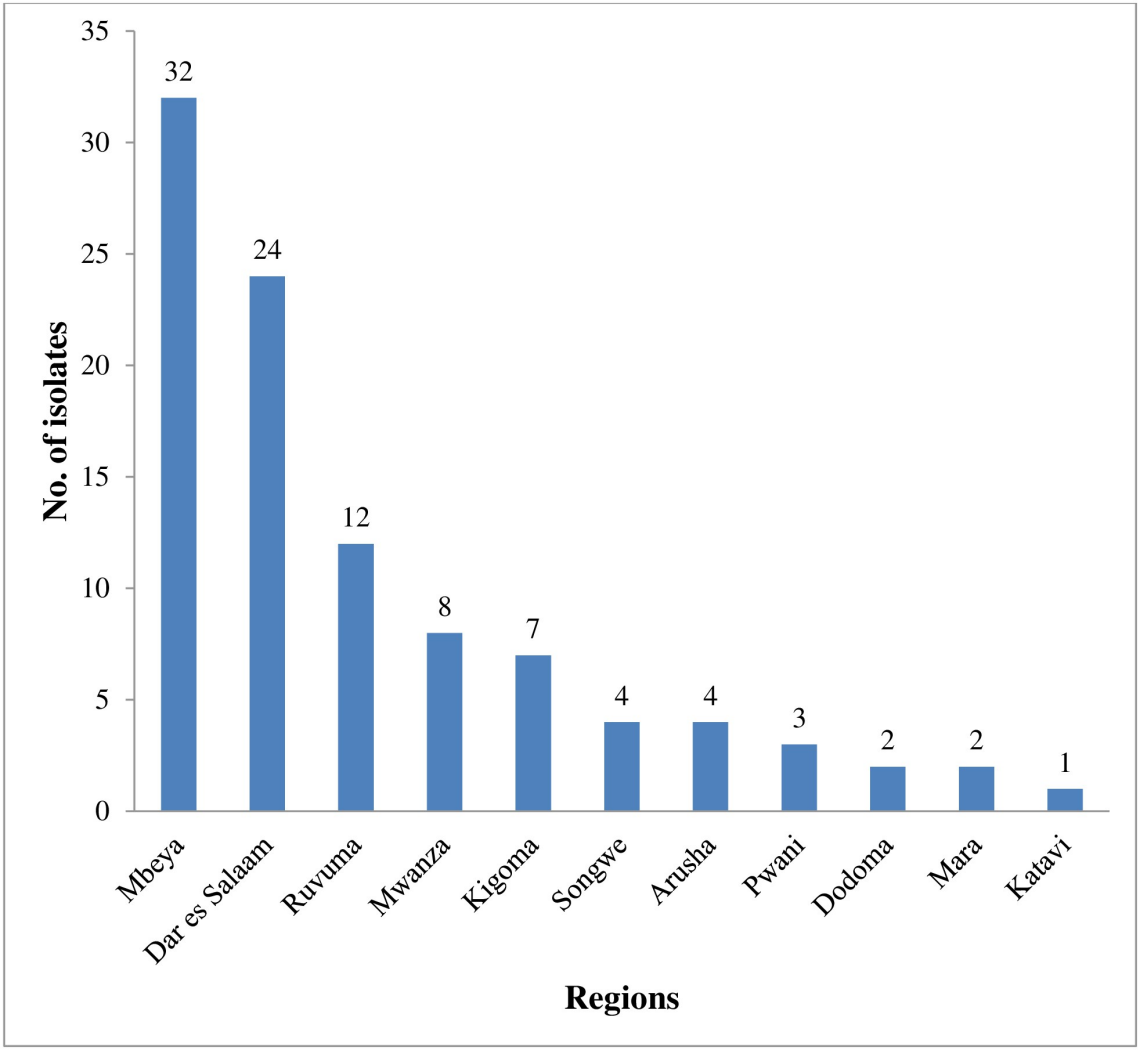
Distribution of laboratory-confirmed *V*.*cholerae* isolates.

A total of 99 of *V*. *cholerae* isolates were investigated; Thirty-two (32.3%) isolates were from Mbeya region with Dar es Salaam contributing 24 (24.2%) of the isolates. Katavi region had only one isolate. All isolates were *V*. *cholerae* serogroup O1, with 97 (98.0%) being Ogawa serotype, and only 2 (2%) were Inaba serotype. Mbeya was the only region that reported both Ogawa and Inaba serotypes (Fig 1).

### Genetic relatedness of *V*. *cholerae* isolates

**Fig 2:**
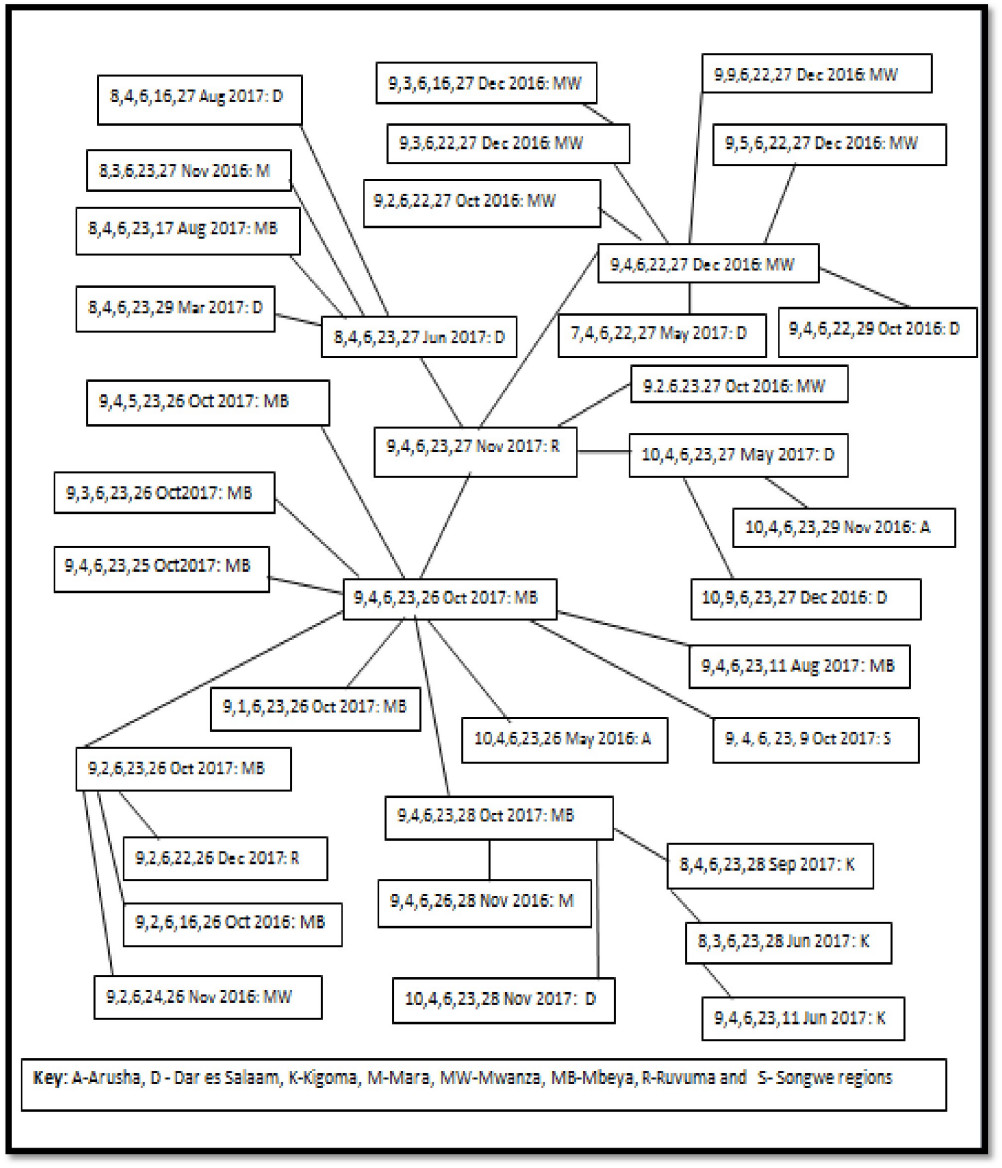
Genetic relatedness between *V*.*cholerae* genotypes in CC1 by MLVA, time, and regions. Five numbers represent genotype; each connecting line shows an allelic change at a single locus of a genotype.

**Fig 3:**
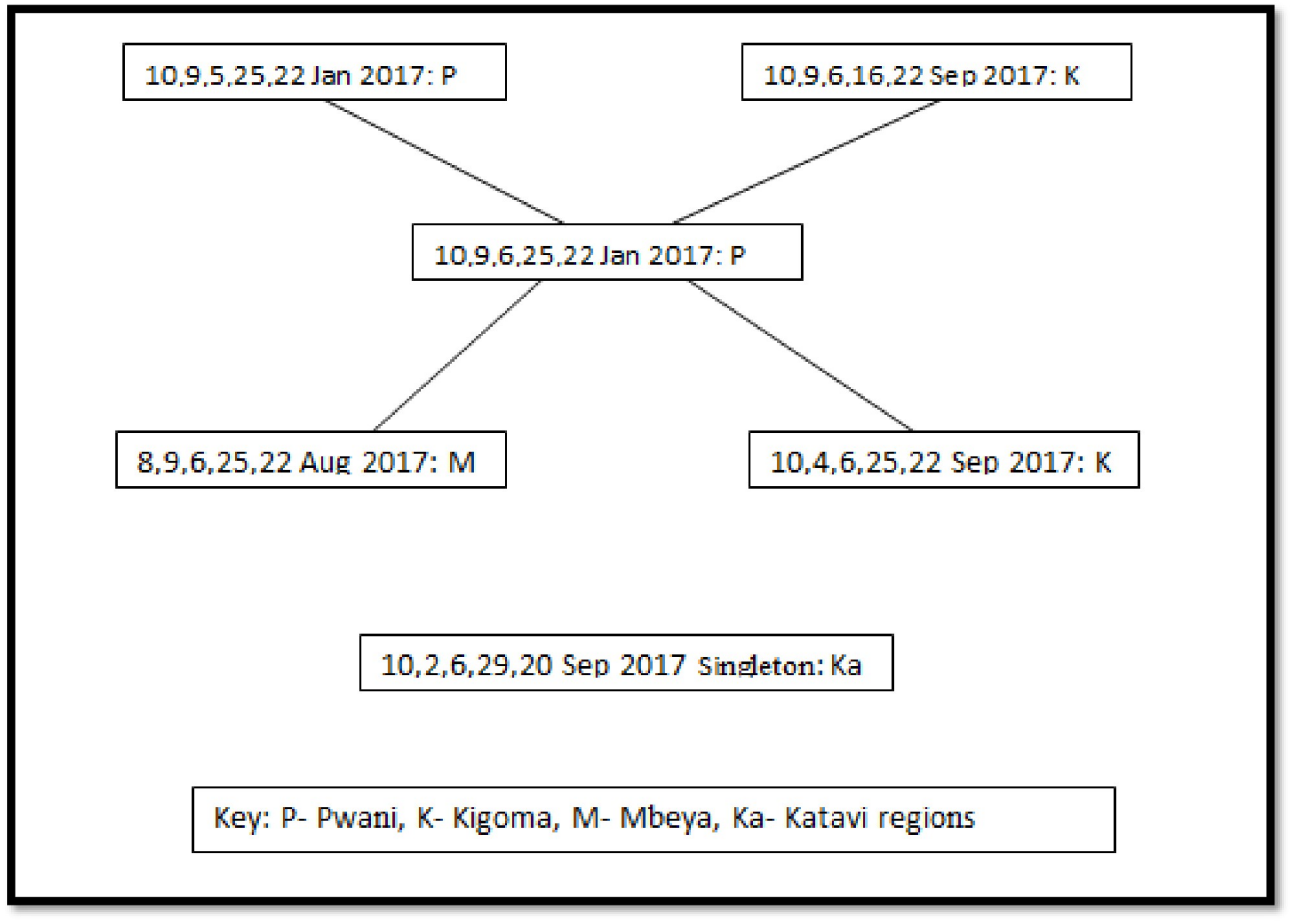
Genetic relatedness between *V*.*cholerae* genotypes in CC2 and one singleton genotype by MLVA, time, and regions. Five numbers represent genotype; each connecting line shows an allelic change at a single locus of a genotype. CC2 represents five genotypes. One singleton genotype that differed at two or more loci to the other genotypes was considered genetically unrelated.

MLVA was conducted to determine the genetic relatedness. Isolates were subjected to MLVA using specific primers of five loci in order of the number of repeats units at each locus. The results showed that isolates were genetically related, with a slight genetic diversity. A total of 42 genotypes were identified; two clonal complexes and one singleton. The clonal complex one (CC1) had 36 (85.7%) genotypes (Fig 2), while CC2 had only five (14.3%) genotypes (Fig 3). One singleton that differed at two or more loci to other genotypes was considered unrelated (Fig 3). Isolates from Dar es Salaam Mwanza, Mbeya and Ruvuma had similar characteristics (Fig 2). The MLVA genotypes based on regions are presented in S2 Table.

### *V*. *cholerae* O1 biotypes and virulence factors

**Fig 4:**
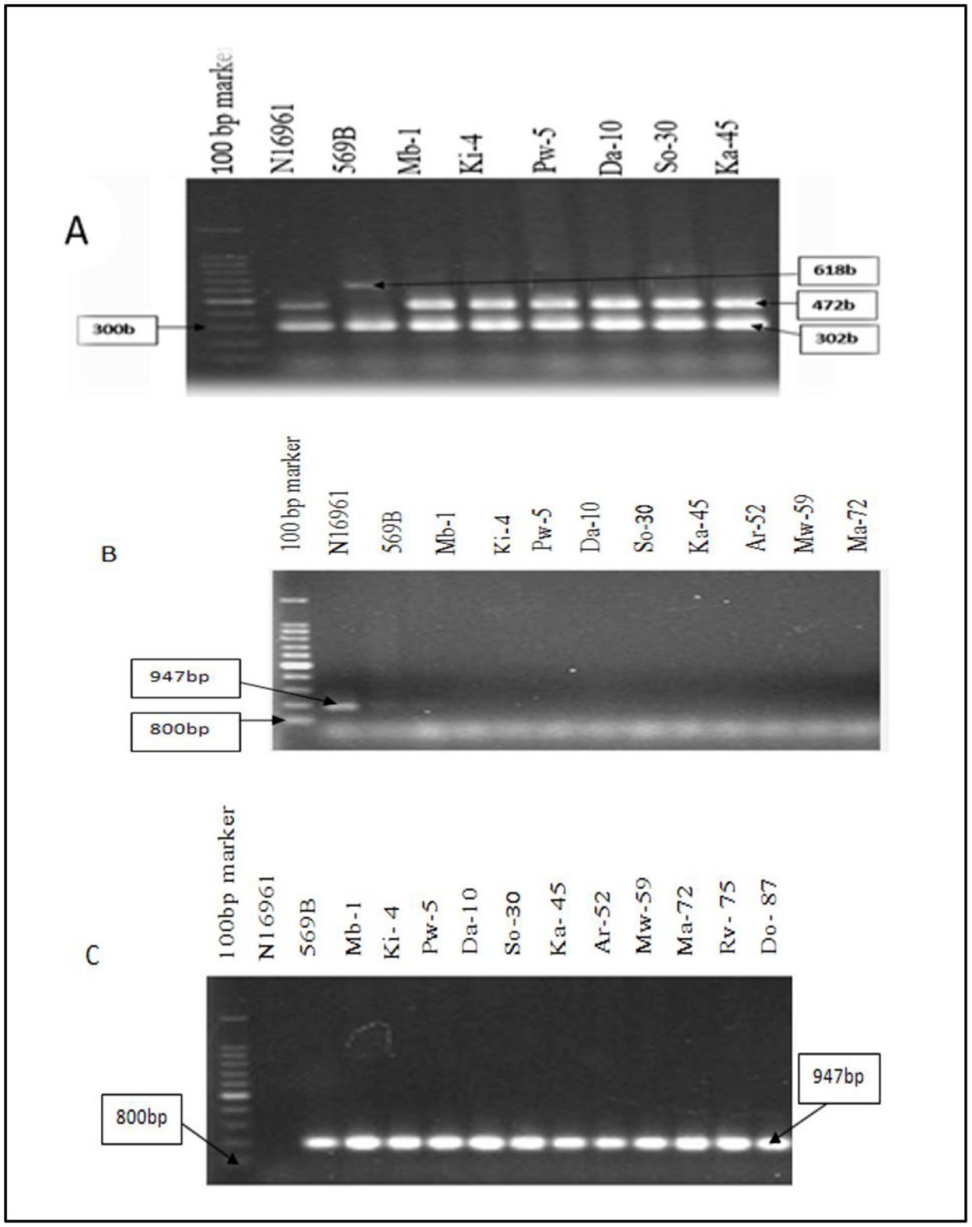
MAMA PCR gel electrophoresis. Examples of electrophoresis results of MAMA PCR products of *V*.*cholerae* O1 strains A: Detection of *ctxA* (302bp) and *tcpA* (472bp) genes. B: Detection of biotype-specific cholera toxin B (*ctxB*) El Tor gene. C: Detection of biotype-specific cholera toxin B *(ctxB)* Classical gene. Positive control (569B) and negative control (N16961) for *ctxB* (El Tor) gene were used. *V*. *cholerae* DNA; Mb-1(Mbeya- 2017), Ki-4 (Kigoma 2017), Pw-5 (Pwani 2017), Da-10 (Dar-es-Salaam- 2017), So-30 (Songwe 2017), Ka-45 (Katavi 2017), Ar-52 (Arusha 2016), Mw-59 (Mwanza 2016), Ma-72 (Mara 2016), Rv-75 (Ruvuma 2017), and Do-87 (Dodoma 2016). The numbers represent the DNA identity.

Table 1 summarises the frequency of virulence factors and Fig 4 presents typical results of electrophoresis of MAMA PCR products. Overall, 79 of the 99 isolates were selected to represent the whole batch of isolates as the remaining 20 were clonally related, using VNTR, to the selected isolates. All 79 strains were positive for the *ctxA* gene that codes for cholera toxin subunit A and *tcpA* (El Tor) that codes for toxin co-regulated pili for intestinal colonization (Fig 4A). All 79 strains tested negative for *ctxB* El Tor gene that encodes for cholera toxin subunit B of the El Tor type (Fig 4B). The *ctxB* gene that code for cholera toxin of the classical biotype was found in all 79 isolates (Fig 4C). The use of *V*.*cholerae* reference strains, N16961 (El Tor biotype), and 569B (classical biotype) generated PCR products which indicated that the primers were working optimally.

**Table 1:** Frequency of toxin and virulence genes detected (n = 79).

Virulence gene	Frequency (%)
Cholera toxin subunit A (*ctxA*)	79 (100)
Toxin co-regulated pili *tcpA* (El Tor)	79 (100)
Toxin co-regulated pili *tcpA* (Classical)	0
Cholera toxin subunit B El Tor (*ctxB*)	0
Cholera toxin subunit B Classical (*ctxB*)	79 (100)

### Antimicrobial susceptibility patternof *V*.*cholerae* O1 strains

All 99 isolates (100%) were sensitive to trimethoprim-sulphamethoxazole, tetracycline, doxycycline, ceftriaxone, and chloramphenicol while 88% were susceptible to ciprofloxacin. Resistance to nalidixic acid, ampicillin, erythromycin, and amoxicillin was 100% (S1 Table).

## Discussion

The current study determined the genetic relatedness, virulence genes, and antimicrobial susceptibility patterns of the *V*.*cholerae* isolated from various regions in Tanzania during the cholera outbreak that occurred between 2016 and 2017. *V*. *cholerae* O1 Ogawa was the predominant serotype. Generally, isolates were genetically related, irrespective of the time of the outbreak. All eleven regions had serotype Ogawa, while serotype Inaba was only detected in Mbeya region. Wide distribution of *V*. *cholerae* O1 Ogawa was reported in 2011 and 2015 outbreaks that occurred in Tanzania Mainland [17] and in 2009 in Zanzibar [18]. On the contrary, a study in Kenya reported Inaba as the predominant serotype [19]. The difference in serotype distribution could be a result of serotype switching. Previous studies indicate that serotype switching frequently occurs in the cholera-endemic area as a result of selective pressure due to immunity acquisition during infection [20, 21].

The current study demonstrated the close genetic relatedness among isolates, causing outbreaks between 2016 and 2017 with a slight genetic diversity in the affected regions. Of the 42 genotypes detected, the isolate that determined the group occurred in Dar es Salaam, spread to Mbeya and Ruvuma regions, and then to other regions. The findings signify that the Dar es Salaam region was the source of the cholera outbreaks that occurred from 2016 to 2017. The current findings differ from those reported in the earlier outbreaks that occurred between 2011 and 2015, where *V*.*cholerae* demonstrated an extensive genetic diversity [17]. This study found El Tor strains harbouring the classical cholera toxin gene, a finding that is similar to reports from other countries like Malaysia, Thailand, Haiti, and India [22–26]. Classical and El Tor are the commonly reported biotypes of *V*.*cholerae* O1, though, other atypical variants of El Tor biotypes exist [5–8]. El Tor strains have extraordinary adaptability and survival capacity in the environment, colonize better in the small intestine and have a more efficient host-to-host transmission than classical strains. Furthermore, El Tor strains can produce a similar cholera toxin to that of classical enhancing virulence [27]. Our findings contribute to the evidence of the persistence of cholera outbreak in our settings. The virulence genes (*ctxA*, *tcpA*, *and ctxB)* of *V*. *cholerae* O1 strain detected in this study are consistent with findings in Malaysia [22]. Moreover, the findings are in line with those from studies done in Zanzibar and Thailand, which reported El Tor variant strains expressing genotypes of the classical biotype that possess *ctxB* [18, 25].

Most *V*. *cholerae* isolates in this study were susceptible to the antibiotics currently used in the treatment of cholera in Tanzania, including doxycycline and ciprofloxacin. However, resistance to fluoroquinolones, including ciprofloxacin, has been reported [19]. Notably, all isolates were susceptible to trimethoprim-sulphamethoxazole, ceftriaxone, and tetracycline. These findings widen alternative antibiotics for cholera treatment as these are among the antibiotics recommended by center for disease control for the management of cholera patients [28]. The isolates demonstrated high resistance (100%) to erythromycin, nalidixic acid, ampicillin, and amoxicillin. Hounmanou et al., reported similar trend of *V*.*cholerae* isolates being phenotypically susceptible to trimethoprim, ciprofloxacin, ceftazidime, tetracycline and chloramphenicol but genotyically resistant to trimethoprim [29]. On the contrary, a study by Urassa et al., conducted twenty years ago reported a high rate of resistance towards trimethoprim-sulphamethoxazole but sensitive to ampicillin, erythromycin, nalidixic acid indicating a change in susceptibility patterns [30]. A situation where phenotypic findings differ from genotypic profile has also been reported elsewhere [31]. The emergence of antibiotic-resistant strains reported in this study calls for periodic monitoring of susceptibility pattern since it has clinical implications for patients, including prolonged illness, extended periods of infectivity, and prolong hospital stay.

## Conclusion

The cholera outbreaks in Tanzania between 2016 and 2017 were mainly caused by clonally related *V*.*cholerae* O1, serotype Ogawa, El tor variant. The outbreaks have a single primary source where the interventions should be focused to stop further spread of the disease. Most isolates were susceptible to the antibiotics currently used to manage cholera cases in Tanzania. However, high resistance rate detected for previously used antibiotics regime calls for continuous monitoring of the trend of antimicrobial susceptibility testing during the outbreaks.

## Supporting information

S1 Table Antimicrobial susceptibility pattern of *V*.*cholerae* O1 strain (n = 99). (PDF)

S2 Table MLVA genotypes of *V*.*cholerae* O1 isolated from Tanzania Mainland, 2016–2017. (PDF)

S1 Raw images (TIF)
