# Supplementary material for: Genetic relatedness, virulence factors and antibiotics susceptibility pattern of Vibrio cholerae isolates from various regions during cholera outbreak in Tanzania
Source: PLoS One. 2022 Mar 25;17(3):e0265868. doi: 10.1371/journal.pone.0265868 (PMC8956160; doi:10.1371/journal.pone.0265868)
Supplement: S1 Table — (PDF) [file pone.0265868.s001.pdf]

S1 Table: Antimicrobial susceptibility pattern of *V.cholerae* O1 strain (n=99)

Antimicrobial drugs	Sensitive (%)	Resistant (%)
Trimethoprim-sulphamethoxazole	100	0
Chloramphenicol	100	0
Tetracycline	100	0
Doxycycline	100	0
Ceftriaxone	100	0
Ciprofloxacin	87.8	12.2
Nalidixic acid	0	100
Erythromycin	0	100
Ampicillin	0	100
Amoxicillin	0	100
