# Supplementary material for: Genetic relatedness, virulence factors and antibiotics susceptibility pattern of Vibrio cholerae isolates from various regions during cholera outbreak in Tanzania
Source: PLoS One. 2022 Mar 25;17(3):e0265868. doi: 10.1371/journal.pone.0265868 (PMC8956160; doi:10.1371/journal.pone.0265868)
Supplement: S2 Table — (PDF) [file pone.0265868.s002.pdf]

S2 Table: MLVA genotypes of *V.cholerae* O1 isolated from Tanzania Mainland, 2016-2017.

MLVA Profile					Genotypes	Clonal complex	Region
7	4	6	22	27	1	1	Dar es Salaam
8	3	6	16	28	2	1	Dar es Salaam
8	3	6	23	28	3	1	Kigoma
8	3	6	23	27	4	1	Mara
8	4	6	23	28	5	1	Kigoma
8	4	6	23	27	6	1	Dar es Salaam
8	4	6	16	27	7	1	Dar es Salaam
8	4	6	23	17	8	1	Mbeya
8	4	6	23	29	9	1	Dar es Salaam
9	1	6	23	26	11	1	Mbeya
9	2	6	23	26	12	1	Mbeya
9	2	6	16	26	13	1	Mbeya
9	2	6	22	27	14	1	Mwanza
9	2	6	23	27	15	1	Mwanza
9	2	6	24	26	16	1	Ruvuma
9	2	6	22	26	17	1	Mwanza
9	3	6	23	26	18	1	Mbeya
9	3	6	22	27	19	1	Mwanza
9	3	6	16	27	20	1	Mwanza
9	4	6	23	26	21	1	Mbeya

9	4	6	23	11	22	1	Mbeya
9	4	6	23	25	23	1	Mbeya
9	4	5	23	26	24	1	Mbeya
9	4	6	23	26	25	1	Mbeya
9	4	6	23	9	26	1	Songwe
9	4	6	23	28	27	1	Mbeya
9	4	6	26	28	28	1	Mara
9	4	6	22	27	29	1	Mwanza
9	4	6	23	27	30	1	Ruvuma
9	4	6	22	29	31	1	Dar es Salaam
9	5	6	22	27	32	1	Mwanza
9	9	6	22	27	33	1	Mwanza
10	4	6	23	26	35	1	Arusha
10	4	6	23	27	36	1	Dar es Salaam
10	4	6	23	28	38	1	Dar es Salaam
10	4	6	23	29	39	1	Arusha
10	9	6	23	27	43	1	Dar es Salaam
8	9	6	25	22	10	2	Mbeya
10	4	6	25	22	37	2	Kigoma
10	9	6	25	22	40	2	Pwani
10	9	6	16	22	41	2	Kigoma
10	9	5	25	22	42	2	Pwani
10	2	6	29	20	34	Singleton	Katavi
