# Supplementary figures and images for: Genetic relatedness, virulence factors and antibiotics susceptibility pattern of Vibrio cholerae isolates from various regions during cholera outbreak in Tanzania
Source: PLoS One. 2022 Mar 25;17(3):e0265868. doi: 10.1371/journal.pone.0265868 (PMC8956160; doi:10.1371/journal.pone.0265868)

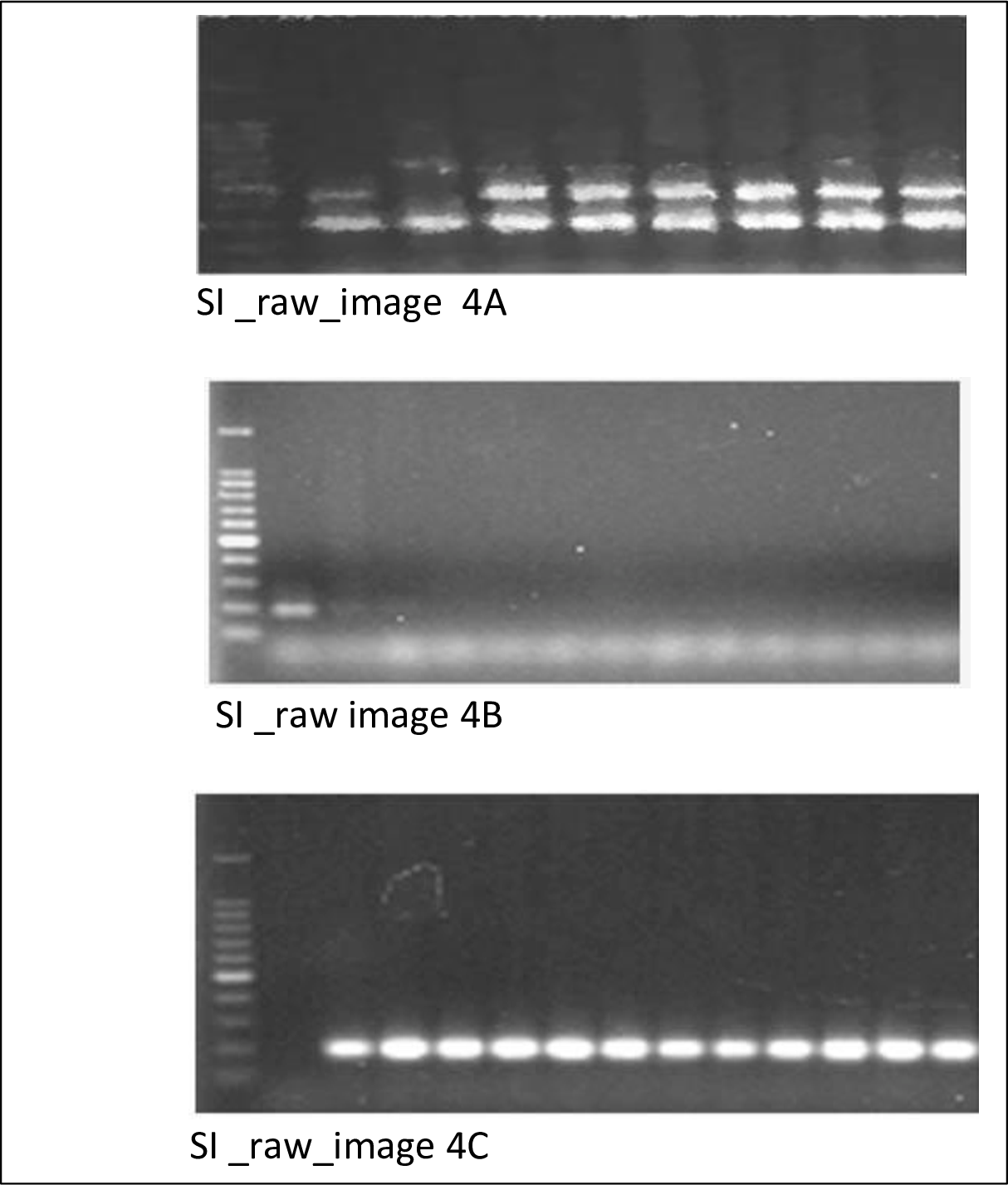

Supplement: S1 Raw images — (TIF) [file pone.0265868.s003.tif]
